# Theory of the Formation of Bone

**Published:** 1855-04

**Authors:** 


*Theory of the Formation of Bone.*—M. Flourens, the justly eminent Professor of Comparative Physiology at the Natural History Museum of Paris, is now giving his usual course of lectures on this important branch of medical studies. From the report of these lectures, given in *L'Union Medicale*, we extract the following interesting facts connected with the theory of the formation of bone.

"The *external* periosteum mainly contributes to the *formation* of bone; but the *internal* periosteum (medullary membrane) is the principal agent in the *absorption* of bone. Around the dead portion of bone an internal periosteum forms, belonging to the new bone, which periosteum seizes, and, as it were, gnaws and corrodes the old bone, and finally causes its absorption. (Does not the purulent matter issuing from the fragments carry off a good deal of osseous detritus?) In general terms, in the healthy bone, the internal periosteum may be looked upon as an apparatus chiefly designed for *resorption.* The mechanism of the development of bone appears to consist in a constant mutation of the parts composing it; hence we have mutation of matter, permanence of form. Buffon very justly said, "The most constant and the least variable fact in nature is the *form* or *mould* of each species, and what is most variable and corruptible is *substance.*" Bonnetus, with all the physiologists of his time, believed that the growth of bone was effected by the deposition of new molecules between the old ones; but we now know that bone is not a fixed and constant substance, but one successively and uninterruptedly formed of superficial layers, which become again re-absorbed. Hence the fallacy of the theory of accumulated germs."

It is now perfectly known and acknowledged that the periosteum may act as the regenerator of bone. We shall just adduce a case of the late Blandin, mentioned in these lectures, which offers a good practical illustration of this theory.

The patient was a young man between twenty-five and thirty years of age, affected with caries of the clavicle. Various means employed to arrest the disease having failed, M. Blandin, encouraged by the experiments of Flourens, resolved to remove the sternal half the bone, which had been most damaged by caries. By careful management he succeeded in removing the bone without injuring the periosteum more than by the necessary longitudinal incision. This done, the acromial end of the bone was, at the request of the patient, carefully examined and found equally diseased. The latter insisted on having that portion also removed, which operation was performed in the same manner as the first. The patient recovered, and having returned to the hospital for another complaint, about eight months after this complete extirpation of the clavicle, it was found that that bone had been completely regenerated. (It is difficult to understand how a bone can be carious and its periosteum perfectly healthy.)—*Ibid.*

